# High-Throughput Proteomics Detection of Novel Splice Isoforms in Human Platelets

**DOI:** 10.1371/journal.pone.0005001

**Published:** 2009-03-24

**Authors:** Karen A. Power, James P. McRedmond, Andreas de Stefani, William M. Gallagher, Peadar Ó Gaora

**Affiliations:** 1 UCD Conway Institute and UCD School of Biomolecular & Biomedical Sciences, UCD Conway Institute, University College Dublin, Belfield, Dublin, Ireland; 2 Biontrack, NovaUCD, Belfield Innovation Park, Dublin, Ireland; 3 UCD Conway Institute and UCD School of Medicine & Medical Sciences, UCD Conway Institute, University College Dublin, Belfield, Dublin, Ireland; University of Cape Town, South Africa

## Abstract

Alternative splicing (AS) is an intrinsic regulatory mechanism of all metazoans. Recent findings suggest that 100% of multiexonic human genes give rise to splice isoforms. AS can be specific to tissue type, environment or developmentally regulated. Splice variants have also been implicated in various diseases including cancer. Detection of these variants will enhance our understanding of the complexity of the human genome and provide disease-specific and prognostic biomarkers. We adopted a proteomics approach to identify exon skip events - the most common form of AS. We constructed a database harboring the peptide sequences derived from all hypothetical exon skip junctions in the human genome. Searching tandem mass spectrometry (MS/MS) data against the database allows the detection of exon skip events, directly at the protein level. Here we describe the application of this approach to human platelets, including the mRNA-based verification of novel splice isoforms of *ITGA2*, *NPEPPS* and *FH*. This methodology is applicable to all new or existing MS/MS datasets.

## Introduction

Since the publication of the human genome sequence, understanding the functional complexity of the genome has become a primary goal of high-throughput experimental research. By definition, AS contributes to proteomic complexity but it has also been suggested that AS is a major driver of phenotypic complexity, though this role remains unproven [1]–[3]. By splicing several combinations of exons into different transcripts, AS generates, from a single gene, multiple isoforms of a protein with potentially diverse functions. Not only has AS been invoked as an explanation for our complexity as a species, detection of splice isoforms has been associated with the cause and progression of certain diseases. Alternative splicing is associated with a wide variety of conditions including bipolar disorder, schizophrenia, cancer, diabetes, multiple sclerosis, cystic fibrosis and asthma (for a review see Wang & Cooper [4]). Splice isoforms may be functionally relevant in disease or may act as biomarkers - indicators of normal or altered biological processes or pharmacological response to a therapeutic intervention [5]. Biomarkers such as disease-specific AS isoforms can serve as indicators of disease susceptibility as well as diagnostic and prognostic markers.

Alternative splicing occurs in many cell types including platelets - hemostatic, anucleate cells derived from megakaryocytes. Although devoid of a nucleus, they retain low levels of mRNA which undergo translation. They have an intact spliceosome and cellular activation of platelets induces splicing of pre-mRNAs including IL-1β [6] and tissue factor (TF) [7]. Platelets are primarily involved in thrombus formation but their functions also extend to pathophysiological processes such as host defense, regulation of vascular tone, inflammation and tumor growth [8]. Splice isoforms in platelets have been implicated in the variable response to aspirin [9] and as possible antithrombotic drug targets [10]. Blood-based biomarker discovery would provide minimally invasive and sensitive detection of disease-associated molecular changes. Disease biomarkers, serving as specific diagnostic signatures of phenotype, could improve drug discovery and facilitate the development of modern, personalized clinical applications.

To date, efforts to detect AS events have relied primarily on sequencing mature mRNA species. The bulk of our knowledge comes from mapping expressed sequence tags (ESTs) to the genome. However, this approach is hindered by the lack of EST coverage with few ESTs sequenced for most genes [11] and the central region of mRNAs inadequately represented. More recently, exon arrays have been developed to determine genome-wide exon expression levels. This technology detects differences in expression across a gene to infer the presence of alternative splicing events, but cannot determine unambiguously what combination of exons is present on a single mRNA. The inference of AS is confounded somewhat by the variable hybridization intensities of neighboring probe sets within a sample and differential gene expression between samples. Ultra high-throughput sequencing addresses some of the problems encountered with previous methods of AS detection [12]. This approach can identify many alternative splice variants if sufficient sequence reads are carried out [13], [14]. As longer sequence reads become available, it will be possible to identify considerable structure flanking a given AS event.

The capacity to discover AS events at the mRNA level is very powerful and mRNAseq has provided evidence for AS occurring in 100% of multi-exonic human genes [13]. It remains unclear how many of the splice isoforms identified are sufficiently stable to result in translation products. Studying the proteome circumvents this issue - a recent study by Tress and coworkers for example, demonstrated the presence of translated AS isoforms in *Drosophila melanogaster* [15]. The development of new, innovative discovery approaches based on protein expression will greatly enhance the existing methodologies.

**Figure 1:**
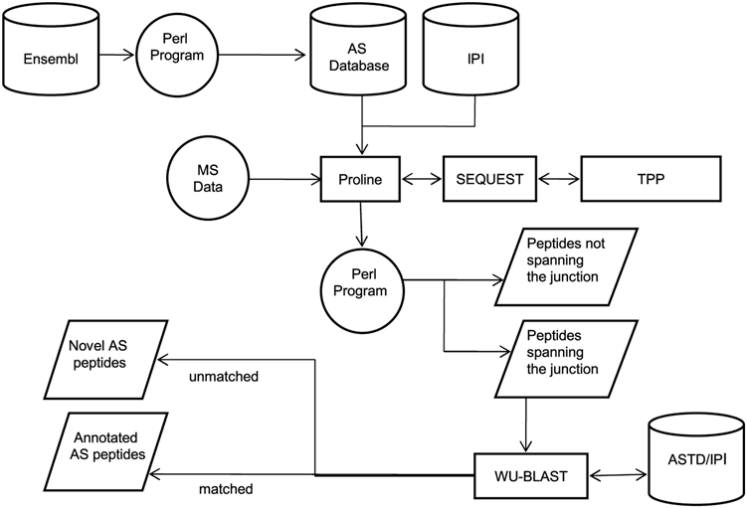
Workflow for the identification of novel exon skip events. A rectangle represents a program, rhombus represents a program output, cylinder represents a data source and circle represents a program input.

Mass spectrometry (MS) has emerged as a highly effective analytical technique capable of detecting vast numbers of peptides in complex mixtures. This is achieved by mapping spectra generated from a MS experiment to a database of known or, more commonly, theoretically derived spectra to infer the peptide sequence. Exon skip splice isoforms are characterized by the peptides spanning the exon-exon junction of a novel splicing event. To detect these peptides, we generate a database containing the theoretical exon-skip junction peptides across a genome. We then use standard MS search tools to identify junction peptides that represent exon skip events in MS/MS spectra by comparison with this database (Fig. 1). Here, we show that this approach can detect novel exon skip events in human platelets and verify a number of these at the mRNA level.

## Results

### Database design

**Figure 2:**
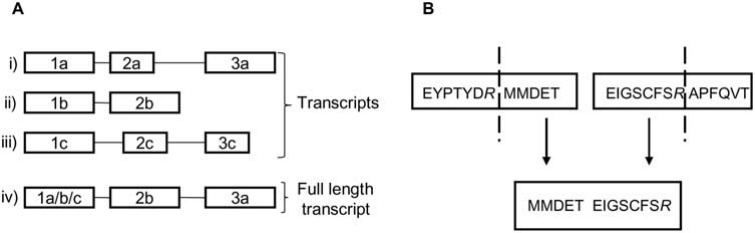
Generation and usage of the SkipE database. (A) Generation of representative transcripts. Each box represents an exon and each line is an intron. i), ii) and iii) represent transcripts from a single gene. iv) shows the full length representative transcript used to generate the junction peptides. (B) Structure of a junction peptide. The top two boxes represent the translated sequences of two separate, non-adjacent exons. The tryptic cleavage sites are represented by dashed vertical bars. The C-terminal sequence of the upstream exon from the final tryptic site is spliced to the N-terminus of the downstream exon and extends to the first tryptic cleavage site of the downstream exon.

The strategy we employed to generate the database (which we call SkipE) is outlined in Figure 1. Transcript and exon data were extracted from Ensembl v46 [16] for all 22,680 annotated human protein-coding genes. To create exon skip junctions *in silico,* a gene containing multiple transcripts was first reduced to a single ‘full length transcript’ (Fig. 2a) as described in Materials and Methods.

All non-contiguous junction peptides in a ‘full length transcript’ were created such that the termini are trypsin cleavage sites (Fig. 2b). It is possible to design a database for other proteolytic enzymes but trypsin is by far the most commonly employed proteinase in proteomics experiments. Combinations of exons yielding junction peptides were constrained by the phase of the exons in order to keep the sequences within the correct reading frame. Phase describes the number of nucleotides upstream of an exon that are used to form a codon so that the length of the exon is a multiple of three. A previous study by Sorek *et al.* [17] showed, using coding sequence information from Genbank, that the majority of orthologous alternatively spliced exons conserved between human and mouse did not endure a frame shift. Furthermore, it is likely that many phase shifting splice events generate transcripts which are degraded *via* nonsense-mediated decay [18]. In order to detect only alternative splice events in which the correct reading frame is maintained, the phase of both exons joined by the alternatively spliced junction was calculated and only those junctions with exons of compatible phase were entered into the database.

Duplicate entries of the same junction peptide mapping to different genes were removed to eliminate ambiguity, since the source of such peptides could not be ascribed to a particular gene. This procedure yielded 307,030 junction peptides for the human genome. Previous genome-based studies, such as 6-frame translation of the genome, result in search spaces that are incompatible with high-throughput approaches. Genome-based methods that reduce the search space complexity, provide a powerful means to identify new protein-coding exons and genes but are not appropriate for direct mapping of exon skips since these junctions are derived from non-contiguous sequences [19]. The database we constructed, subject to the constraints described, generates a search space appropriate for the high-throughput MS/MS methods in use today and into the future. Further details on the composition of the human, mouse and rat databases are provided (Table S1).

The skipE database is in FASTA format and therefore suitable for use with any of the major search engines; in this case we employed SEQUEST [20] combined with PeptideProphet and ProteinProphet for statistical validation of identifications [21]. We chose a cutoff score of 0.9, a commonly used cutoff in MS/MS experiments [22], for both tools. We then determined which junction-spanning peptides are novel and those which were previously described by comparing peptide sequences with the Alternative Splice Transcript Database (ASTD) [23]–[25] and the International Protein Index database (IPI) [26] using WU-BLAST (http://blast.wustl.edu). This also filters out junction peptides which are identical to sequences within “canonical” isoforms, whether they occur at exon boundaries or elsewhere.

### Identification of platelet proteins and AS peptides

Platelet mass spectra were collected and compared with both the IPI and SkipE databases to identify peptides. The number of peptides and proteins identified in each database are shown in table S2. SEQUEST searching against IPI identified 6,292 unique peptides representing 1,122 unique proteins in the samples with a ProteinProphet probability score of P>0.9. Since the SkipE database harbors peptide rather than protein sequences, ProteinProphet is inappropriate. Therefore, spectra identified by comparison with SkipE were validated using a PeptideProphet probability cut-off of 0.9 resulting in 1,297 unique protein identifications. Of these, 359 were represented by more than a single occurrence of the peptide in the dataset.

**Figure 3:**
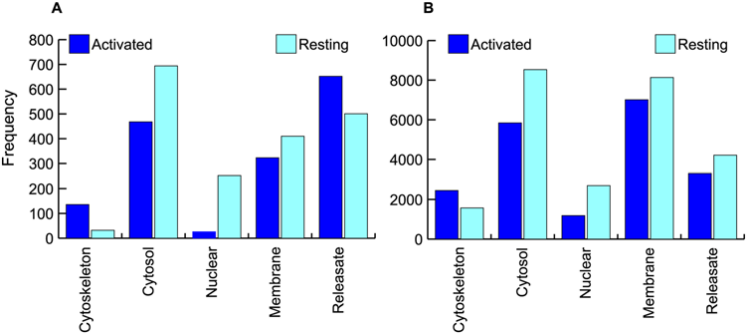
Characteristics of the exon skip events detected in human platelets. (A) and (B) describe the distribution of the SkipE and IPI peptides respectively, across the different subcellular compartments for both resting and activated platelet samples.

The spatial distribution of AS identifications closely mirrors that of the IPI data with the exception of the releasate (Fig. 3a, b). In this case, more skips were found in the activated than in the resting samples for the AS data. Although the activation step was very brief, this may indicate a tendency towards diversification of the exported proteome in response to platelet activation. Functionally, this would be advantageous since these cells must interact with the *milieu* and other cell types but cannot mount a transcriptomic response to stimuli. All identified proteins in both SkipE and IPI data were mapped to KEGG pathways using Pathway-Express [27] (Table S3 and S4). In a typical MS/MS data analysis, protein identifications rely on multiple peptide identifications for any given protein. Since SkipE harbors isolated peptide sequences, we decided to focus further experiments on those AS events for which evidence of cognate gene expression was also obtained in the IPI analysis. Therefore, we constructed a list of 89 genes which represents the intersection of the AS and IPI datasets (Table 1).

**Table 1:** Description of the 89 genes identified in both SkipE and IPI.

Gene Symbol	Ensembl Gene ID	Exon ID 1	Exon ID 2	Description
ACLY	ENSG00000131473	ENSE00000898911	ENSE00000898879	ATP-citrate synthase
ACOX1	ENSG00000161533	ENSE00001222343	ENSE00001117984	Acyl-coenzyme A oxidase 1, peroxisomal
ACTN4	ENSG00000130402	ENSE00000895798	ENSE00000895787	Alpha-actinin-4
ALOX12	ENSG00000108839	ENSE00000905333	ENSE00000887238	Arachidonate 12-lipoxygenase, 12S-type
AMPD2	ENSG00000116337	ENSE00001153152	ENSE00000913099	AMP deaminase 2
AP1B1	ENSG00000100280	ENSE00000652055	ENSE00000652051	AP-1 complex subunit beta-1
APOB	ENSG00000084674	ENSE00000932268	ENSE00000718984	Apolipoprotein B-100 precursor
APOL1	ENSG00000100342	ENSE00000935990	ENSE00001369317	Apolipoprotein-L1 precursor
ARHGEF7	ENSG00000102606	ENSE00000686804	ENSE00000686825	Rho guanine nucleotide exchange factor 7
ARHGEF7	ENSG00000102606	ENSE00001236980	ENSE00000686833	Rho guanine nucleotide exchange factor 7
ATIC	ENSG00000138363	ENSE00001363573	ENSE00001146950	Bifunctional purine biosynthesis protein PURH
ATP5C1	ENSG00000165629	ENSE00001481323	ENSE00001094820	ATP synthase gamma chain, mitochondrial precursor
C2	ENSG00000204364	ENSE00001467298	ENSE00001467293	Complement C2 precursor
C21orf33	ENSG00000160221	ENSE00001506662	ENSE00001506660	ES1 protein homolog, mitochondrial precursor
C3	ENSG00000125730	ENSE00001053527	ENSE00000858107	Complement C3 precursor
C3	ENSG00000125730	ENSE00001053551	ENSE00000858104	Complement C3 precursor
CCT5	ENSG00000150753	ENSE00001082664	ENSE00001082663	T-complex protein 1 subunit epsilon
CD109	ENSG00000156535	ENSE00001144336	ENSE00001144250	CD109 antigen precursor
CD109	ENSG00000156535	ENSE00001144243	ENSE00001084417	CD109 antigen precursor
CLTC	ENSG00000141367	ENSE00000948100	ENSE00000948105	Clathrin heavy chain 1
CLTCL1	ENSG00000070371	ENSE00000596272	ENSE00001343357	Clathrin heavy chain 2
COL14A1	ENSG00000187955	ENSE00001022732	ENSE00001090753	Collagen alpha-1
COL14A1	ENSG00000187955	ENSE00000702894	ENSE00001476378	Collagen alpha-1
COPB1	ENSG00000129083	ENSE00000886038	ENSE00000703797	Coatomer subunit beta
COPB2	ENSG00000184432	ENSE00001322263	ENSE00001311447	Coatomer subunit beta
CP	ENSG00000047457	ENSE00001008190	ENSE00000779559	Ceruloplasmin precursor
CSE1L	ENSG00000124207	ENSE00000845497	ENSE00000845507	Exportin-2
CYFIP1	ENSG00000068793	ENSE00000883355	ENSE00000883353	Cytoplasmic FMR1-interacting protein 1
DCTN1	ENSG00000204843	ENSE00001261315	ENSE00001199793	Dynactin-1
ENO1	ENSG00000074800	ENSE00000739712	ENSE00000738913	Alpha-enolase
FAM62A	ENSG00000139641	ENSE00000939452	ENSE00000939471	Protein FAM62A
FH	ENSG00000091483	ENSE00000961691	ENSE00001069123	Fumarate hydratase, mitochondrial precursor
FLII	ENSG00000177731	ENSE00001289389	ENSE00001289270	Protein flightless-1 homolog.
FLNA	ENSG00000196924	ENSE00000678331	ENSE00000868362	Filamin-A
GLUD1	ENSG00000148672	ENSE00000986500	ENSE00000986506	Glutamate dehydrogenase 1, mitochondrial precursor
GPD2	ENSG00000115159	ENSE00000924640	ENSE00001188495	Glycerol-3-phosphate dehydrogenase, mitochondrial precursor
GUCY1A3	ENSG00000164116	ENSE00001231799	ENSE00001081588	Guanylate cyclase soluble subunit alpha-3
HD	ENSG00000197386	ENSE00000854949	ENSE00000854981	Huntington disease protein
HD	ENSG00000197386	ENSE00000854965	ENSE00001251513	Huntington disease protein
HD	ENSG00000197386	ENSE00000854958	ENSE00000854991	Huntington disease protein
HD	ENSG00000197386	ENSE00000854979	ENSE00000855002	Huntington disease protein
HERC2	ENSG00000128731	ENSE00000672196	ENSE00001275912	Probable E3 ubiquitin-protein ligase HERC2
HERC2	ENSG00000128731	ENSE00000672179	ENSE00001275876	Probable E3 ubiquitin-protein ligase HERC2
HERC2	ENSG00000128731	ENSE00000908550	ENSE00000908562	Probable E3 ubiquitin-protein ligase HERC2
HK1	ENSG00000156515	ENSE00001145338	ENSE00001276961	Hexokinase-1
HSD17B4	ENSG00000133835	ENSE00001143964	ENSE00000972282	Peroxisomal multifunctional enzyme type 2
HSD17B4	ENSG00000133835	ENSE00001143927	ENSE00000972282	Peroxisomal multifunctional enzyme type 2
HSD17B4	ENSG00000133835	ENSE00001169924	ENSE00001144014	Peroxisomal multifunctional enzyme type 2
HSD17B4	ENSG00000133835	ENSE00001143964	ENSE00001143927	Peroxisomal multifunctional enzyme type 2
HYOU1	ENSG00000149428	ENSE00001195270	ENSE00000990519	150 kDa oxygen-regulated protein precursor
IQGAP2	ENSG00000145703	ENSE00000971759	ENSE00001030776	Ras GTPase-activating-like protein IQGAP2.
ITGA2	ENSG00000164171	ENSE00001082079	ENSE00001082066	Integrin alpha-2 precursor
ITGA2	ENSG00000164171	ENSE00001082085	ENSE00001082079	Integrin alpha-2 precursor
ITGB3	ENSG00000056345	ENSE00000947489	ENSE00000735016	Integrin beta-3 precursor
ITIH2	ENSG00000151655	ENSE00001415117	ENSE00001395332	Inter-alpha-trypsin inhibitor heavy chain H2 precursor
ITPR1	ENSG00000150995	ENSE00001072653	ENSE00001122088	Inositol 1,4,5-trisphosphate receptor type 1
KIF5B	ENSG00000170759	ENSE00001163763	ENSE00001163716	Kinesin heavy chain
KRT16	ENSG00000186832	ENSE00001118312	ENSE00001118295	Keratin, type I cytoskeletal 16
KTN1	ENSG00000126777	ENSE00001292736	ENSE00000867340	Kinectin
LCP2	ENSG00000043462	ENSE00000769281	ENSE00000812799	Lymphocyte cytosolic protein 2
LRRFIP2	ENSG00000093167	ENSE00000825531	ENSE00000760563	Leucine-rich repeat flightless-interacting protein 2
LTBP1	ENSG00000049323	ENSE00000932484	ENSE00000932488	Latent-transforming growth factor beta-binding protein, isoform 1L precursor
LTBP1	ENSG00000049323	ENSE00000932483	ENSE00001006678	Latent-transforming growth factor beta-binding protein, isoform 1L precursor
LTBP1	ENSG00000049323	ENSE00000932485	ENSE00000744639	Latent-transforming growth factor beta-binding protein, isoform 1L precursor
LTBP1	ENSG00000049323	ENSE00000809557	ENSE00000744639	Latent-transforming growth factor beta-binding protein, isoform 1L precursor
MACF1	ENSG00000127603	ENSE00001041391	ENSE00001079474	Microtubule-actin cross-linking factor 1, isoforms 1/2/3/5
MACF1	ENSG00000127603	ENSE00001408360	ENSE00001218066	Microtubule-actin cross-linking factor 1, isoforms 1/2/3/5
MACF1	ENSG00000127603	ENSE00001411283	ENSE00001218029	Microtubule-actin cross-linking factor 1, isoforms 1/2/3/5
MACF1	ENSG00000127603	ENSE00001411283	ENSE00001041391	Microtubule-actin cross-linking factor 1, isoforms 1/2/3/5
MMRN1	ENSG00000138722	ENSE00001003940	ENSE00001003943	Multimerin-1 precursor
MTCH2	ENSG00000109919	ENSE00000714864	ENSE00001267224	Mitochondrial carrier homolog 2
MTHFD1	ENSG00000100714	ENSE00000658410	ENSE00000658424	C-1-tetrahydrofolate synthase, cytoplasmic
MTHFD1	ENSG00000100714	ENSE00000658406	ENSE00000658420	C-1-tetrahydrofolate synthase, cytoplasmic
MYH4	ENSG00000141048	ENSE00000907666	ENSE00000907657	Myosin-4
NID2	ENSG00000087303	ENSE00000854715	ENSE00000657316	Nidogen-2 precursor
NID2	ENSG00000087303	ENSE00000657316	ENSE00000854708	Nidogen-2 precursor
NPEPPS	ENSG00000141279	ENSE00001138170	ENSE00001138132	Puromycin-sensitive aminopeptidase
NRBP1	ENSG00000115216	ENSE00000809167	ENSE00000733215	Nuclear receptor-binding protein.
OGDH	ENSG00000105953	ENSE00000681534	ENSE00000681548	2-oxoglutarate dehydrogenase E1 component, mitochondrial precursor
PDIA5	ENSG00000065485	ENSE00001149277	ENSE00001353839	Protein disulfide-isomerase A5 precursor
PICALM	ENSG00000073921	ENSE00000742961	ENSE00001376469	Phosphatidylinositol-binding clathrin assembly protein
PIP5K2A	ENSG00000150867	ENSE00000996551	ENSE00000996552	Phosphatidylinositol-4-phosphate 5-kinase type-2 alpha
PKHD1L1	ENSG00000205038	ENSE00001477427	ENSE00001477413	fibrocystin L
PKHD1L1	ENSG00000205038	ENSE00001477417	ENSE00001477394	fibrocystin L
PKHD1L1	ENSG00000205038	ENSE00001477471	ENSE00001477421	fibrocystin L
PKHD1L1	ENSG00000205038	ENSE00001477455	ENSE00001477439	fibrocystin L
PKHD1L1	ENSG00000205038	ENSE00001477437	ENSE00001477347	fibrocystin L
PKHD1L1	ENSG00000205038	ENSE00001477474	ENSE00001477449	fibrocystin L
PLEC1	ENSG00000178209	ENSE00001244151	ENSE00001244041	Plectin-1
PLEC1	ENSG00000178209	ENSE00001244070	ENSE00001295392	Plectin-1
PLG	ENSG00000122194	ENSE00000828808	ENSE00001315450	Plasminogen precursor
PLXDC2	ENSG00000120594	ENSE00001137970	ENSE00000996527	Plexin domain-containing protein 2 precursor
PROS1	ENSG00000184500	ENSE00001142430	ENSE00001142413	Vitamin K-dependent protein S precursor.
PSMC6	ENSG00000100519	ENSE00000657442	ENSE00000657448	26S protease regulatory subunit S10B
PTPN18	ENSG00000072135	ENSE00000436095	ENSE00000776192	Tyrosine-protein phosphatase non-receptor type 18
RAB8A	ENSG00000167461	ENSE00001113277	ENSE00001277163	Ras-related protein Rab-8A
RASA3	ENSG00000185989	ENSE00001334941	ENSE00001334928	Ras GTPase-activating protein 3
RTN2	ENSG00000125744	ENSE00000858227	ENSE00000858223	Reticulon-2
SNX17	ENSG00000115234	ENSE00000734775	ENSE00000734780	Sorting nexin-17
SNX17	ENSG00000115234	ENSE00000962998	ENSE00000734785	Sorting nexin-17
SPTBN1	ENSG00000115306	ENSE00001036038	ENSE00001036017	Spectrin beta chain, brain 1
SRC	ENSG00000197122	ENSE00001390472	ENSE00000661882	Proto-oncogene tyrosine-protein kinase Src
STOM	ENSG00000148175	ENSE00000983575	ENSE00001262522	Erythrocyte band 7 integral membrane protein
THBS1	ENSG00000137801	ENSE00000883758	ENSE00000883772	Thrombospondin-1 precursor
TMEM33	ENSG00000109133	ENSE00001489658	ENSE00000712706	Transmembrane protein 33
TMOD3	ENSG00000138594	ENSE00001170748	ENSE00001102815	Tropomodulin-3
TPD52L2	ENSG00000101150	ENSE00000663594	ENSE00001391722	Tumor protein D54
UBASH3B	ENSG00000154127	ENSE00001014167	ENSE00001014158	Suppressor of T-cell receptor signaling 1
UBE1L	ENSG00000182179	ENSE00001305417	ENSE00001306981	Ubiquitin-activating enzyme E1 homolog
UGCGL1	ENSG00000136731	ENSE00001148961	ENSE00001206051	UDP-glucose:glycoprotein glucosyltransferase 1 precursor
UGP2	ENSG00000169764	ENSE00001189522	ENSE00001165982	UTP–glucose-1-phosphate uridylyltransferase 2
UNC13D	ENSG00000092929	ENSE00001227797	ENSE00001406672	Unc-13 homolog D
UNC13D	ENSG00000092929	ENSE00001227615	ENSE00001430590	Unc-13 homolog D
USP14	ENSG00000101557	ENSE00001208659	ENSE00001252715	Ubiquitin carboxyl-terminal hydrolase 14
VPS13A	ENSG00000197969	ENSE00001024130	ENSE00000803886	Vacuolar protein sorting-associated protein 13A
VPS13A	ENSG00000197969	ENSE00001024085	ENSE00000708339	Vacuolar protein sorting-associated protein 13A
VPS13A	ENSG00000197969	ENSE00000708190	ENSE00000708458	Vacuolar protein sorting-associated protein 13A
VPS13A	ENSG00000197969	ENSE00001171911	ENSE00000803905	Vacuolar protein sorting-associated protein 13A
VPS13A	ENSG00000197969	ENSE00001024110	ENSE00000708298	Vacuolar protein sorting-associated protein 13A
VPS13A	ENSG00000197969	ENSE00001024141	ENSE00001024126	Vacuolar protein sorting-associated protein 13A
VPS13A	ENSG00000197969	ENSE00001024130	ENSE00000707929	Vacuolar protein sorting-associated protein 13A
VPS13C	ENSG00000129003	ENSE00001124918	ENSE00000885044	Vacuolar protein sorting-associated protein 13C.
VPS13C	ENSG00000129003	ENSE00001124912	ENSE00001364815	Vacuolar protein sorting-associated protein 13C.
VPS13C	ENSG00000129003	ENSE00000449795	ENSE00001380396	Vacuolar protein sorting-associated protein 13C.
VPS13C	ENSG00000129003	ENSE00000885045	ENSE00001368990	Vacuolar protein sorting-associated protein 13C.
VPS13C	ENSG00000129003	ENSE00000885061	ENSE00001484949	Vacuolar protein sorting-associated protein 13C.
VPS13C	ENSG00000129003	ENSE00000885061	ENSE00000885051	Vacuolar protein sorting-associated protein 13C.
WAS	ENSG00000015285	ENSE00000669947	ENSE00001255082	Wiskott-Aldrich syndrome protein
WDR44	ENSG00000131725	ENSE00000899838	ENSE00000899846	WD repeat protein 44

The Gene symbol, Ensembl gene and exon identifiers and the gene descriptions are listed for all 129 junctions found in 89 genes. The exon identifiers one and two indicate the exons involved in the junction peptide identified in SkipE.

### Verification of splice variants at mRNA level

**Figure 4:**
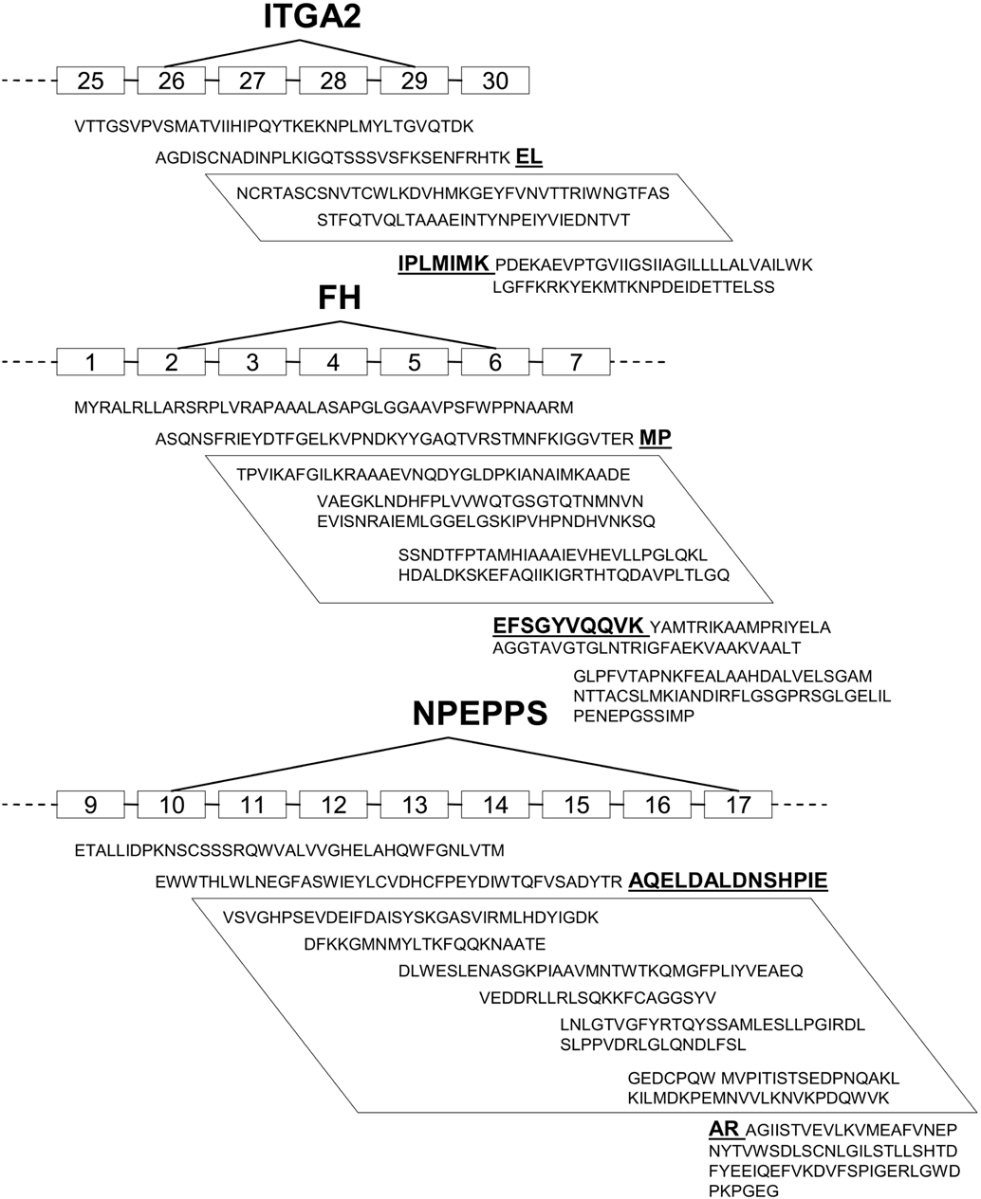
Exon skip events verified at mRNA level. Each numbered box represents an exon and the position in the gene. The skip event is indicated by the diagonal lines. The parallelograms enclose the portion of amino acid sequence that is absent from the novel splice isoform. The bold and underlined form the junction peptides.

We confirmed the presence of several mRNA species encoding previously undescribed exon skip events by RT-PCR and sequencing of the products. We chose 3 junctions identified in the SkipE data for which evidence of protein expression was obtained in the IPI search (Fig. 4 **)**. The proteins chosen were integrin alpha 2 or platelet glycoprotein Ia (*ITGA2*), fumarate hydratase (*FH*) and puromycin-sensitive aminopeptidase (*NPEPPS*). These proteins represent different compartments and perform various roles in the cell.

*ITGA2* forms part of a platelet collagen receptor, involved in the initial adhesion of platelets to extracellular matrix exposed at sites of endothelial injury, such as atherosclerotic lesions [28], [29]. Splice variants may be functionally significant: a platelet-specific splice variant may allow some tissue specific functions, while polymorphic variations in *ITGA2* are associated with risk of thrombotic stroke [30]. The junction peptide we identified, which was formed by splicing exon 26 to exon 29, occurred 3 times in the SkipE data and 16 peptides were present for this protein in the IPI data. This splicing event results in the deletion of 68 amino acids proximal to the single transmembrane domain on the extracellular surface, far from any reported ligand-binding domains. Similar changes in the length of the ‘stalk’ of the platelet adhesion receptor GPIb are reported to affect the ability of platelets to adhere at high flow rates [31].

*FH* is a Krebs' cycle enzyme which is located in the cytosol or can be transported to the mitochondrion and has been shown to act as a tumor suppressor [32]. The *FH* junction under study was formed by splicing exon 2 to exon 6 and was identified 5 times with 7 different peptides identified in the IPI data.

The final protein selected, *NPEPPS*, is a puromycin-sensitive aminopeptidase, common in brain and immune tissues. NPEPPS may play a role in cell development and cell cycle-regulating proteolysis [33]. The *NPEPPS* junction identified was created *via* the splicing of exon 10 to exon 17 and occurred 4 times while 4 peptides were identified in IPI sequences.

**Figure 5:**
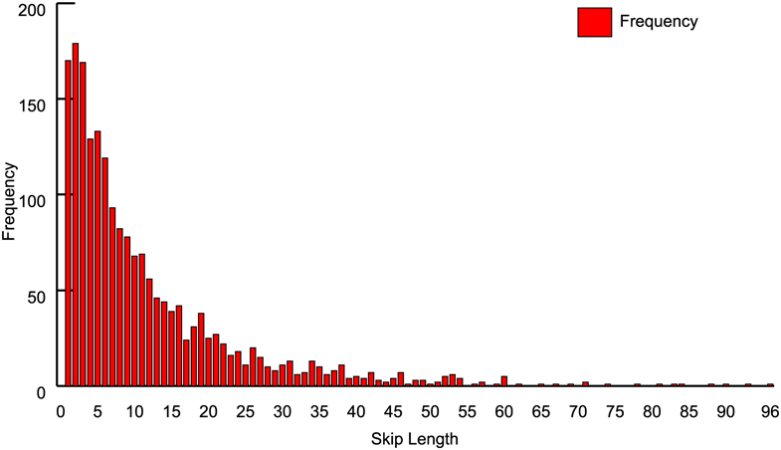
Frequency distribution of exon skip lengths identified in the SkipE database. The number of occurrences of each skip length identified is shown.

The NPEPPS event was the longest skip we investigated, removing 6 exons. Interestingly, skips of up to 96 exons were observed – the distribution of skip lengths shown in Fig. 5 is highly reminiscent of that observed by Sultan *et al*. in mRNAseq data [14]. Such long skips remain to be verified (perhaps by the use of 2-dimensional gel separation followed by Western blotting and/or MS), as the number of other potential AS events in genes exhibiting long range AS gives rise to multiple PCR products (data not shown). Primer pairs specific to the exons involved in each junction generated multiple or ambiguous products with a predominant band migrating at the “canonical” amplicon length. It is likely that the AS message is present in relatively small amounts and is out-competed by the canonical isoform in PCR. Therefore, we designed primers to span the novel junctions and paired them with compatible reverse primers providing a skip-specific PCR primer pair (Table 2).

**Table 2:** Design of exon-junction-spanning PCR primers.

Gene	Junction Peptide	Junction Primer									Product Length (bp)
*NPEPPS*	AQELDALDNSHPIEAR	T	CCT	ATT	GAA	–	GCT	CGA	GCT	G	200
			P	I	E		A	R			
*FH*	MPEFSGYVQQVK	AA	CGC	ATG	CCA	–	GAA	TTT	AGT	G	165
				M	P		E	F	S		
*ITGA2*	ELIPLMIMKPDEK	CC	AAA	GAA	TTG	–	ATT	CCC	CTG	A	115
				E	L		I	P	L		

The gene symbol, junction peptide sequence, junction primer and product length are shown. The junction primer column indicates, with a dash, the exon-exon boundary.

**Figure 6:**
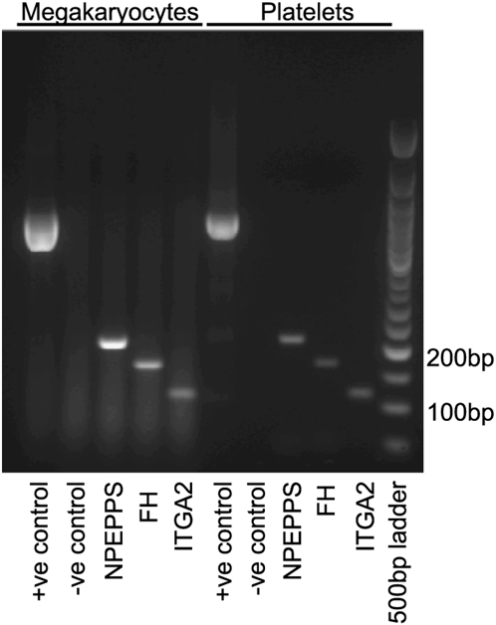
Agarose gel electrophoresis of PCR products. Lanes 1–5 show products amplified from megakaryocyte cDNA; samples in lanes 6–10 contained template from platelets. Molecular size markers are in lane 11.

PCR products of the expected sizes were observed in each case with cDNA derived both from platelets and from their precursors, megakaryocytes. The bands derived from platelet cDNA were excised and the sequence verified that the predicted products were obtained. It can be seen from Figure 6 that the megakaryocyte template produced a greater quantity of the amplicon in each case, reflective of the availability of template rather than an increased proportion of AS message in these cells.

## Discussion

Our findings demonstrate that many exon skip events, which have not been previously described, occur in platelets. These events have been found in a novel high-throughput fashion. The approach described is compatible with existing MS/MS software solutions accessible to the scientific community. We have shown that, while these events were found computationally, using a proteomics platform, we selected and verified three of them at the transcriptomic level by PCR and sequencing.

It is notable that the overlap of proteins, identified in the AS and IPI databases, is relatively low – just 89 genes were represented by peptides in both datasets. In common with many other high-throughput experimental approaches such as yeast two-hybrid and protein interaction networking [34], [35], MS/MS proteomics experiments suffer from a lack of completeness - that is, coverage of the proteome is neither absolute nor unbiased. The completeness of proteomics experiments is increased by high-throughput approaches although approximately 10 repetitions of a multidimensional protein identification technology (MudPIT) experiment are required to reach 95% analytical completeness [36], [37]. The proteins identified in any given experiment will be constrained by a number of factors including expression level and presence of proteotypic peptides [38]. In the case of splice isoforms, these will not necessarily correspond to the ‘canonical’ isoforms. Therefore, although, in this experiment we used IPI-based detection of protein expression to filter potential targets for verification, it is clear that not all genes displaying AS will also be detected as canonical isoforms and *vice versa*. Although we applied a relatively strict cutoff of 0.9 to the SkipE hits, given the fact that they are subject to only PeptideProphet and not ProteinProphet validation, it is possible there are more false positives in the SkipE data than the IPI results. Ultra high throughput mRNAseq verification of high numbers of skip events detected in proteomics data will demonstrate the synergy derived from the combination of high-throughput techniques and these datasets will provide mutual cross validation.

It appears that the novel splice events detected in this study were most likely inherited from the precursor rather than being specific to the platelet. It will be interesting to determine the distribution of these events in a variety of cell types and tissues across different organisms. It will also be of interest to determine whether any of the exon skip events occur specifically in the platelet since it is known that splicing can occur in these cells, despite the absence of a nucleus. While exon skips are the most common type of AS event described to date [13], [14], [39], [40], several other splicing patterns occur during transcription including alternative 5′ and 3′ splice sites and intron retention. These events require a different approach to detection in proteomics data. Clearly, intron-specific peptides could be incorporated into the SkipE database, though this would considerably increase the database size. A parallel intron peptide database would be a feasible approach. Alternative 5′ or 3′ splice sites on the other hand are not amenable to detection in this manner and require an alternative approach.

In conclusion, we have developed a novel database, suitable for the detection of alternative splicing in mass spectrometry data and shown that it can detect AS events in a platelet MS/MS dataset. The approach described augments current methodologies. Detection of AS directly at the protein level avoids any requirement for amplification steps and indicates that the events detected are indeed expressed. Millions of spectra, which are already available in both public and private repositories, can be reanalyzed using this database. As label-free quantitation tools are incorporated into proteomics pipelines, the added value becomes even greater as isoforms can be compared at the expression level within and between samples. Again, this approach is applicable to the vast repositories of data already gathered as well as to all new samples. The application of this methodology will rapidly give us new insights into AS throughout a range of tissue types and biological states. Since AS events have previously been associated with particular diseases, the approach described here will allow the discovery of disease-specific biomarkers at the splice isoform level. As the proteome is the network most closely related to the biological phenotype, the potential to discover clinically relevant biomarkers related to diagnosis, prognosis or susceptibility is immense, impacting on all levels of clinical practice and drug development.

### Note added in proof

During the review process a similar database development was described by Mo *et al*. [41].

## Materials and Methods

### Platelet MS/MS data acquisition

Platelets were prepared as previously described in McRedmond *et al.* [42] and incubated at 37°C with stirring. One sample was activated by the addition of 5 µM thrombin receptor activating peptide for 5 minutes. Resting and activated samples were separated into subcellular compartments using a ProteoExtract subcellular proteome extraction kit (Merck Biosciences, Nottingham, UK). The manufacturer's protocol was modified to ensure separation of platelet pellets from supernatants and to allow the recovery of released platelet proteins. This procedure yields a ‘nuclear’ fraction, which is artefactual when applied to platelets. Fractions from resting and thrombin receptor activating peptide-activated platelets were separated by SDS-PAGE; gel lanes were cut into 32 slices and digested with trypsin. Peptides were separated by single-dimension reverse-phase liquid chromatography and analysed using an LTQ ion trap mass spectrometer (Thermo-Finnigan, San Jose, CA) [43].

### Public data repositories

Ensembl version 46 was used to obtain all protein coding genes and sequences, along with their associated exon predictions for the human, mouse and rat genomes. Previously annotated AS events in our dataset were filtered out by comparing sequences with ASTD version 1.1 and IPI version 3.16 using Washington University basic local alignment search tool (WU-BLAST) version 2.0, applying the pam30 substitution matrix.

### Database development

Transcript and exon data were extracted, *via* the Perl-API, from Ensembl v46 for all annotated genes in each of the human, mouse and rat genomes. For each species, a separate database was generated. Briefly, a standard “full-length transcript” containing, for each exon position along the transcript, the longest predicted exon sequence was generated. This procedure yields a single, representative, “standard” transcript from which to design junction peptides. The junction peptides are the derived peptide sequences that span exon-exon junctions from the most C-terminal protease site in the upstream exon to the most N-terminal protease site in the downstream exon. In this case, we used trypsin as the protease. Only the junctions of non-consecutive exons were included in the database and the content was further constrained by only including junctions in which phase was maintained between exons. The fasta files for all three species are publicly available online at http://bioinformatics.ucd.ie/SkipE.

### MS/MS data analysis

All MS/MS data analyses were carried out using the Proline proteomics platform (Biontrack, Dublin http://www.biontrack.com). Spectra were compared against databases using SEQUEST [20]. Validation of peptides and proteins was carried out using the transproteomics pipeline tools PeptideProphet and ProteinProphet [21], respectively, and filtered with a cut-off of P>0.9.

### RNA isolation

RNA from platelet and the megakaryocytic cell line Meg-01 was isolated as previously described [42] and reverse-transcribed into cDNA using standard techniques.

### Validation

PCR and sequencing was carried out to validate the alternative splicing events. All primer synthesis and sequencing was carried out by MWG biotech (http://www.eurofinsdna.com/). Primer sequences for *ITGA2* were, forward CAAAGAATTGATTCCCCTGA and reverse TGCAACCAGAGCTAACAGCA. *NPEPPS* forward primer is TCCTATTGAAGCTCGAGCTG and reverse CAGCCCAGTCTCTCCCCTAT and *FH* forward primer is AACGCATGCCAGAATTTAGTG and reverse is CCACTTTTGCAGCAACCTTT. The PCR reactions were made up as follows; 8 µl 5× GoTaq buffer, 1 µl Taq polymerase, 2 µl 4 mM dNTPs (Promega), 22 µl H2O, 2 µl primers and 1.5 µl template. The following PCR conditions were used: 2 minutes of denaturation at 94°C followed by 40 cycles of 30 seconds denaturing at 94°C, 30 seconds annealing at 55°C for *NPEPPS* and 58°C for *FH* and *ITGA2* and a 90 second extension at 72°C followed by incubation at 4°C. Products were separated on 2% agarose gels. Positive control was integrin *ITGA2*B (α2B), a known abundant platelet glycoprotein. Negative control was a no-template RT reaction.

## Supporting Information

Table S1 Characteristics of the contents and constraints applied to create the species-specific SkipE databases.
(0.03 MB DOC)

Table S2 Numbers of platelet peptide and protein identifications in IPI and SkipE databases
(0.03 MB DOC)

Table S3 KEGG annotations for all of the 89 genes found to be alternatively spliced and represented in the IPI data. In total, 32 pathways were found. These pathways are sorted by impact factor, a probabilistic term which is calculated from the number of genes in the input file, the size of the reference chip (U133 plus2.0), the number of input genes that are on a given pathway and the number of the pathway genes represented on the reference chip.
(0.08 MB DOC)

Table S4 KEGG annotations for all the genes found in IPI. In total, 78 pathways were found. These pathways are sorted by impact factor, a probabilistic term which is calculated from the number of genes in the input file, the size of the reference chip (U133 plus2.0), the number of input genes that are on a given pathway and the number of the pathway genes represented on the reference chip.
(0.17 MB DOC)
