# Supplementary material for: High-Throughput Proteomics Detection of Novel Splice Isoforms in Human Platelets
Source: PLoS One. 2009 Mar 24;4(3):e5001. doi: 10.1371/journal.pone.0005001 (PMC2654914; doi:10.1371/journal.pone.0005001)
Supplement: Table S1 — Characteristics of the contents and constraints applied to create the species-specific SkipE databases. (0.03 MB DOC) [file pone.0005001.s001.doc]

|  | **HUMAN** | **MOUSE** | **RAT** |
| --- | --- | --- | --- |
| Protein-coding genes | 22,680 | 23,954 | 22,993 |
| Protein-coding exons | 184,632 | 181,949 | 185,467 |
| Junction Peptides | 307,030 | 307,788 | 318,703 |
| Duplicate peptide entries subsequently removed to avoid ambiguity | 35,383 | 20,644 | 19,201 |
| Number of junctions removed due to an exon beginning or ending in R/K | 51,882 | 48,291 | 51,986 |
| Number of junctions removed due to the new intermediate amino acid being R/K or stop codon | 31,748 | 28,853 | 31,192 |
| Average Junction Peptide length | 22.25 | 22.42 | 22.03 |

**Table S1.** Characteristics of the contents and constraints applied to create the species-specific SkipE databases.
