# Supplementary material for: High-Throughput Proteomics Detection of Novel Splice Isoforms in Human Platelets
Source: PLoS One. 2009 Mar 24;4(3):e5001. doi: 10.1371/journal.pone.0005001 (PMC2654914; doi:10.1371/journal.pone.0005001)
Supplement: Table S2 — Numbers of platelet peptide and protein identifications in IPI and SkipE databases (0.03 MB DOC) [file pone.0005001.s002.doc]

|  | **Peptides** | **Unique Peptides** | **Protein ids** |
| --- | --- | --- | --- |
| IPI | 14,667 | 6,292 | 1,122 |
| SkipE | 2,353 | 1,952 | 1,297 |

**Table S2.** Numbers of platelet peptide and protein identifications in IPI and SkipE databases
