# Supplementary material for: High-Throughput Proteomics Detection of Novel Splice Isoforms in Human Platelets
Source: PLoS One. 2009 Mar 24;4(3):e5001. doi: 10.1371/journal.pone.0005001 (PMC2654914; doi:10.1371/journal.pone.0005001)
Supplement: Table S3 — KEGG annotations for all of the 89 genes found to be alternatively spliced and represented in the IPI data. In total, 32 pathways were found. These pathways are sorted by impact factor, a probabilistic term which is calculated from the number of genes in the input file, the size of the reference chip (U133 plus2.0), the number of input genes that are on a given pathway and the number of the pathway genes represented on the reference chip. (0.08 MB DOC) [file pone.0005001.s003.doc]

| **Rank** | **Pathway Name** | **Impact Factor** | **#Genes in Pathway** | **#Input Genes in Pathway** | **#Pathway Genes on Chip** | **%Input Genes in Pathway** | **%Pathway Genes in Input** | **p-value** |
| --- | --- | --- | --- | --- | --- | --- | --- | --- |
| 1 | Regulation of actin cytoskeleton | 10.413 | 218 | 7 | 205 | 7.955 | 3.211 | 3.00E-05 |
| 2 | Complement and coagulation cascades | 8.505 | 69 | 4 | 67 | 4.545 | 5.797 | 2.02E-04 |
| 3 | Focal adhesion | 8.456 | 200 | 6 | 197 | 6.818 | 3 | 2.13E-04 |
| 4 | Huntington''s disease | 8.144 | 30 | 3 | 30 | 3.409 | 10 | 2.91E-04 |
| 5 | Adherens junction | 5.472 | 75 | 3 | 75 | 3.409 | 4 | 0.0042 |
| 6 | ECM-receptor interaction | 5.09 | 87 | 3 | 86 | 3.409 | 3.448 | 0.00616 |
| 7 | Gap junction | 4.875 | 98 | 3 | 93 | 3.409 | 3.061 | 0.00764 |
| 8 | Tight junction | 4.37 | 136 | 3 | 112 | 3.409 | 2.206 | 0.01265 |
| 9 | Long-term depression | 3.152 | 76 | 2 | 76 | 2.273 | 2.632 | 0.04278 |
| 10 | Hematopoietic cell lineage | 2.972 | 88 | 2 | 84 | 2.273 | 2.273 | 0.05119 |
| 11 | TGF-beta signaling pathway | 2.951 | 90 | 2 | 85 | 2.273 | 2.222 | 0.05228 |
| 12 | Polyunsaturated fatty acid biosynthesis | 2.833 | 16 | 1 | 14 | 1.136 | 6.25 | 0.05882 |
| 13 | GnRH signaling pathway | 2.718 | 97 | 2 | 97 | 2.273 | 2.062 | 0.06598 |
| 14 | Cell Communication | 2.258 | 138 | 2 | 127 | 2.273 | 1.449 | 0.10451 |
| 15 | Bladder cancer | 1.793 | 42 | 1 | 42 | 1.136 | 2.381 | 0.16639 |
| 16 | $hsa05131$ | 1.672 | 53 | 1 | 48 | 1.136 | 1.887 | 0.18781 |
| 17 | Pathogenic Escherichia coli infection | 1.672 | 53 | 1 | 48 | 1.136 | 1.887 | 0.18781 |
| 18 | PPAR signaling pathway | 1.391 | 70 | 1 | 66 | 1.136 | 1.429 | 0.24886 |
| 19 | Epithelial cell signaling in Helicobacter pylori infection | 1.378 | 68 | 1 | 67 | 1.136 | 1.471 | 0.25211 |
| 20 | p53 signaling pathway | 1.365 | 68 | 1 | 68 | 1.136 | 1.471 | 0.25535 |
| 21 | Renal cell carcinoma | 1.353 | 69 | 1 | 69 | 1.136 | 1.449 | 0.25858 |
| 22 | Long-term potentiation | 1.353 | 69 | 1 | 69 | 1.136 | 1.449 | 0.25858 |
| 23 | VEGF signaling pathway | 1.34 | 71 | 1 | 70 | 1.136 | 1.408 | 0.26179 |
| 24 | Fc epsilon RI signaling pathway | 1.281 | 77 | 1 | 75 | 1.136 | 1.299 | 0.27765 |
| 25 | Phosphatidylinositol signaling system | 1.259 | 77 | 1 | 77 | 1.136 | 1.299 | 0.2839 |
| 26 | Small cell lung cancer | 1.157 | 87 | 1 | 87 | 1.136 | 1.149 | 0.31436 |
| 27 | ErbB signaling pathway | 1.157 | 87 | 1 | 87 | 1.136 | 1.149 | 0.31436 |
| 28 | T cell receptor signaling pathway | 1.111 | 93 | 1 | 92 | 1.136 | 1.075 | 0.32911 |
| 29 | Leukocyte transendothelial migration | 0.961 | 117 | 1 | 111 | 1.136 | 0.855 | 0.38233 |
| 30 | Natural killer cell mediated cytotoxicity | 0.882 | 132 | 1 | 123 | 1.136 | 0.758 | 0.41377 |
| 31 | Calcium signaling pathway | 0.634 | 175 | 1 | 174 | 1.136 | 0.571 | 0.53066 |
| 32 | MAPK signaling pathway | 0.397 | 262 | 1 | 256 | 1.136 | 0.382 | 0.67214 |

**Table S3**. KEGG annotations for all of the 89 genes found to be alternatively spliced and represented in the IPI data. In total, 32 pathways were found. These pathways are sorted by impact factor, a probabilistic term which is calculated from the number of genes in the input file, the size of the reference chip (U133 plus2.0), the number of input genes that are on a given pathway and the number of the pathway genes represented on the reference chip.
