# Supplementary material for: High-Throughput Proteomics Detection of Novel Splice Isoforms in Human Platelets
Source: PLoS One. 2009 Mar 24;4(3):e5001. doi: 10.1371/journal.pone.0005001 (PMC2654914; doi:10.1371/journal.pone.0005001)
Supplement: Table S4 — KEGG annotations for all the genes found in IPI. In total, 78 pathways were found. These pathways are sorted by impact factor, a probabilistic term which is calculated from the number of genes in the input file, the size of the reference chip (U133 plus2.0), the number of input genes that are on a given pathway and the number of the pathway genes represented on the reference chip. (0.17 MB DOC) [file pone.0005001.s004.doc]

| **Rank** | **Pathway Name** | **Impact Factor** | **#Genes in Pathway** | **#Input Genes in Pathway** | **#Pathway Genes on Chip** | **%Input Genes in Pathway** | **%Pathway Genes in Input** | **p-value** |
| --- | --- | --- | --- | --- | --- | --- | --- | --- |
| 1 | Regulation of actin cytoskeleton | 30.54 | 218 | 46 | 205 | 4.946 | 21.101 | 5.50E-14 |
| 2 | Focal adhesion | 27.64 | 200 | 39 | 197 | 4.194 | 19.5 | 9.91E-13 |
| 3 | $hsa05131$ | 25.91 | 53 | 20 | 48 | 2.151 | 37.736 | 5.61E-12 |
| 4 | Pathogenic Escherichia coli infection | 25.91 | 53 | 20 | 48 | 2.151 | 37.736 | 5.61E-12 |
| 5 | Complement and coagulation cascades | 25.71 | 69 | 26 | 67 | 2.796 | 37.681 | 6.83E-12 |
| 6 | Leukocyte transendothelial migration | 25.41 | 117 | 26 | 111 | 2.796 | 22.222 | 9.24E-12 |
| 7 | Proteasome | 24.91 | 22 | 12 | 20 | 1.29 | 54.545 | 1.52E-11 |
| 8 | Tight junction | 22.38 | 136 | 24 | 112 | 2.581 | 17.647 | 1.92E-10 |
| 9 | Gap junction | 20.8 | 98 | 21 | 93 | 2.258 | 21.429 | 9.24E-10 |
| 10 | SNARE interactions in vesicular transport | 14.85 | 38 | 11 | 36 | 1.183 | 28.947 | 3.55E-07 |
| 11 | Cell Communication | 13.6 | 138 | 20 | 127 | 2.151 | 14.493 | 1.24E-06 |
| 12 | Epithelial cell signaling in Helicobacter pylori infection | 13.36 | 68 | 14 | 67 | 1.505 | 20.588 | 1.58E-06 |
| 13 | Neurodegenerative Diseases | 12.53 | 30 | 9 | 29 | 0.968 | 30 | 3.63E-06 |
| 14 | Huntington''s disease | 12.21 | 30 | 9 | 30 | 0.968 | 30 | 4.98E-06 |
| 15 | Amyotrophic lateral sclerosis (ALS) | 12.16 | 17 | 7 | 17 | 0.753 | 41.176 | 5.25E-06 |
| 16 | Insulin signaling pathway | 11.31 | 138 | 19 | 135 | 2.043 | 13.768 | 1.23E-05 |
| 17 | Cholera | 11.14 | 42 | 10 | 42 | 1.075 | 23.81 | 1.45E-05 |
| 18 | Adherens junction | 10.37 | 75 | 13 | 75 | 1.398 | 17.333 | 3.14E-05 |
| 19 | Long-term potentiation | 9.708 | 69 | 12 | 69 | 1.29 | 17.391 | 6.08E-05 |
| 20 | Antigen processing and presentation | 9.419 | 89 | 12 | 71 | 1.29 | 13.483 | 8.12E-05 |
| 21 | ECM-receptor interaction | 8.903 | 87 | 13 | 86 | 1.398 | 14.943 | 1.36E-04 |
| 22 | PPAR signaling pathway | 8.614 | 70 | 11 | 66 | 1.183 | 15.714 | 1.82E-04 |
| 23 | MAPK signaling pathway | 8.113 | 262 | 25 | 256 | 2.688 | 9.542 | 3.00E-04 |
| 24 | VEGF signaling pathway | 8.08 | 71 | 11 | 70 | 1.183 | 15.493 | 3.10E-04 |
| 25 | Alzheimer''s disease | 7.947 | 22 | 6 | 22 | 0.645 | 27.273 | 3.54E-04 |
| 26 | Apoptosis | 7.784 | 84 | 12 | 84 | 1.29 | 14.286 | 4.16E-04 |
| 27 | Fc epsilon RI signaling pathway | 7.47 | 77 | 11 | 75 | 1.183 | 14.286 | 5.70E-04 |
| 28 | Long-term depression | 7.355 | 76 | 11 | 76 | 1.183 | 14.474 | 6.40E-04 |
| 29 | Axon guidance | 7.201 | 128 | 15 | 128 | 1.613 | 11.719 | 7.46E-04 |
| 30 | Renal cell carcinoma | 6.81 | 69 | 10 | 69 | 1.075 | 14.493 | 0.0011 |
| 31 | Natural killer cell mediated cytotoxicity | 6.525 | 132 | 14 | 123 | 1.505 | 10.606 | 0.00147 |
| 32 | Cell adhesion molecules (CAMs) | 5.161 | 133 | 13 | 128 | 1.398 | 9.774 | 0.00573 |
| 33 | B cell receptor signaling pathway | 4.967 | 64 | 8 | 62 | 0.86 | 12.5 | 0.00697 |
| 34 | GnRH signaling pathway | 4.338 | 97 | 10 | 97 | 1.075 | 10.309 | 0.01306 |
| 35 | Calcium signaling pathway | 4.289 | 175 | 15 | 174 | 1.613 | 8.571 | 0.01372 |
| 36 | Hematopoietic cell lineage | 4.249 | 88 | 9 | 84 | 0.968 | 10.227 | 0.01428 |
| 37 | T cell receptor signaling pathway | 3.709 | 93 | 9 | 92 | 0.968 | 9.677 | 0.02451 |
| 38 | p53 signaling pathway | 3.351 | 68 | 7 | 68 | 0.753 | 10.294 | 0.03505 |
| 39 | ErbB signaling pathway | 3.101 | 87 | 8 | 87 | 0.86 | 9.195 | 0.045 |
| 40 | Prion diseases | 3.087 | 1 | 1 | 1 | 0.108 | 100 | 0.04565 |
| 41 | Colorectal cancer | 2.421 | 85 | 7 | 84 | 0.753 | 8.235 | 0.08887 |
| 42 | Small cell lung cancer | 2.279 | 87 | 7 | 87 | 0.753 | 8.046 | 0.10237 |
| 43 | Pancreatic cancer | 2.155 | 73 | 6 | 73 | 0.645 | 8.219 | 0.11586 |
| 44 | Polyunsaturated fatty acid biosynthesis | 2.026 | 16 | 2 | 14 | 0.215 | 12.5 | 0.13191 |
| 45 | Dentatorubropallidoluysian atrophy (DRPLA) | 1.912 | 15 | 2 | 15 | 0.215 | 13.333 | 0.1478 |
| 46 | mTOR signaling pathway | 1.796 | 51 | 4 | 47 | 0.43 | 7.843 | 0.16591 |
| 47 | Parkinson''s disease | 1.711 | 20 | 2 | 17 | 0.215 | 10 | 0.1806 |
| 48 | Renin-angiotensin system | 1.711 | 17 | 2 | 17 | 0.215 | 11.765 | 0.1806 |
| 49 | TGF-beta signaling pathway | 1.65 | 90 | 6 | 85 | 0.645 | 6.667 | 0.19202 |
| 50 | Wnt signaling pathway | 1.497 | 149 | 9 | 146 | 0.968 | 6.04 | 0.22383 |
| 51 | Cell cycle | 1.411 | 115 | 7 | 111 | 0.753 | 6.087 | 0.24398 |
| 52 | Chronic myeloid leukemia | 1.324 | 76 | 5 | 76 | 0.538 | 6.579 | 0.26617 |
| 53 | Type I diabetes mellitus | 1.291 | 44 | 3 | 40 | 0.323 | 6.818 | 0.27512 |
| 54 | Phosphatidylinositol signaling system | 1.291 | 77 | 5 | 77 | 0.538 | 6.494 | 0.27498 |
| 55 | Protein export | 1.165 | 11 | 1 | 8 | 0.108 | 9.091 | 0.31192 |
| 56 | Dorso-ventral axis formation | 1.047 | 28 | 2 | 27 | 0.215 | 7.143 | 0.35106 |
| 57 | Olfactory transduction | 0.875 | 31 | 2 | 31 | 0.215 | 6.452 | 0.41679 |
| 58 | Endometrial cancer | 0.854 | 52 | 3 | 52 | 0.323 | 5.769 | 0.4258 |
| 59 | Non-small cell lung cancer | 0.798 | 54 | 3 | 54 | 0.323 | 5.556 | 0.45013 |
| 60 | Melanogenesis | 0.728 | 102 | 5 | 100 | 0.538 | 4.902 | 0.48289 |
| 61 | Asthma | 0.693 | 30 | 1 | 0 | 0.108 | 3.333 | 0.5 |
| 62 | Autoimmune thyroid disease | 0.693 | 53 | 2 | 0 | 0.215 | 3.774 | 0.5 |
| 63 | Glioma | 0.572 | 64 | 3 | 64 | 0.323 | 4.688 | 0.56428 |
| 64 | Prostate cancer | 0.57 | 89 | 4 | 87 | 0.43 | 4.494 | 0.5658 |
| 65 | Bladder cancer | 0.549 | 42 | 2 | 42 | 0.215 | 4.762 | 0.57743 |
| 66 | Type II diabetes mellitus | 0.527 | 44 | 2 | 43 | 0.215 | 4.545 | 0.59033 |
| 67 | Taste transduction | 0.466 | 53 | 2 | 46 | 0.215 | 3.774 | 0.62726 |
| 68 | Adipocytokine signaling pathway | 0.454 | 72 | 3 | 71 | 0.323 | 4.167 | 0.635 |
| 69 | Acute myeloid leukemia | 0.352 | 57 | 2 | 53 | 0.215 | 3.509 | 0.70327 |
| 70 | Hedgehog signaling pathway | 0.338 | 57 | 2 | 54 | 0.215 | 3.509 | 0.713 |
| 71 | Thyroid cancer | 0.298 | 29 | 1 | 29 | 0.108 | 3.448 | 0.74229 |
| 72 | Glycan structures - degradation | 0.282 | 30 | 1 | 30 | 0.108 | 3.333 | 0.75407 |
| 73 | Glycan structures - biosynthesis 1 | 0.254 | 120 | 4 | 115 | 0.43 | 3.333 | 0.77571 |
| 74 | Toll-like receptor signaling pathway | 0.053 | 102 | 2 | 101 | 0.215 | 1.961 | 0.94836 |
| 75 | Melanoma | 0.037 | 71 | 1 | 71 | 0.108 | 1.408 | 0.96396 |
| 76 | Jak-STAT signaling pathway | 0.031 | 153 | 3 | 150 | 0.323 | 1.961 | 0.96991 |
| 77 | Cytokine-cytokine receptor interaction | 0.015 | 259 | 5 | 238 | 0.538 | 1.931 | 0.98557 |
| 78 | Neuroactive ligand-receptor interaction | 0 | 254 | 2 | 251 | 0.215 | 0.787 | 0.9999 |

**Table S4**. KEGG annotations for all the genes found in IPI. In total, 78 pathways were found. These pathways are sorted by impact factor, a probabilistic term which is calculated by the number of the samples in the input file, the size of the reference chip (U133 plus2.0), the number of input samples that are on the pathway and the number of the pathway genes that are on the reference chip.
